# Axl receptor tyrosine kinase is a regulator of apolipoprotein E

**DOI:** 10.1186/s13041-020-00609-1

**Published:** 2020-05-04

**Authors:** Wenchen Zhao, Jianjia Fan, Iva Kulic, Cheryl Koh, Amanda Clark, Johan Meuller, Ola Engkvist, Samantha Barichievy, Carina Raynoschek, Ryan Hicks, Marcello Maresca, Qi Wang, Dean G. Brown, Alvin Lok, Cameron Parro, Jerome Robert, Hsien-Ya Chou, Andrea M. Zuhl, Michael W. Wood, Nicholas J. Brandon, Cheryl L. Wellington

**Affiliations:** 1 Department of Pathology and Laboratory Medicine, Djavad Mowafaghian Centre for Brain Health, University of British Columbia, 2215 Wesbrook Mall, Vancouver, British Columbia V6T 1Z3 Canada; 2 Mechanistic Biology & Profiling, Discovery Sciences, R&D, AstraZeneca, Boston, USA; 3 Mechanistic Biology & Profiling, Discovery Sciences, R&D, AstraZeneca, Gothenburg, Sweden; 4 Discovery Biology, Discovery Sciences, R&D, AstraZeneca, Gothenburg, Sweden; 5 Discovery Biology, Discovery Sciences, R&D, AstraZeneca, Boston, USA; 6 Neuroscience, BioPharmaceuticals R&D, AstraZeneca, Boston, USA; 7 Hit Discovery, Discovery Sciences, R&D, AstraZeneca, Gothenburg, Sweden; 8 Hit Discovery, Discovery Sciences, R&D, AstraZeneca, Boston, USA

**Keywords:** Apolipoprotein E, AXL, Astrocyte, Alzheimer’s disease

## Abstract

Alzheimer’s disease (AD), the leading cause of dementia, is a chronic neurodegenerative disease. Apolipoprotein E (apoE), which carries lipids in the brain in the form of lipoproteins, plays an undisputed role in AD pathophysiology. A high-throughput phenotypic screen was conducted using a CCF-STTG1 human astrocytoma cell line to identify small molecules that could upregulate apoE secretion. AZ7235, a previously discovered Axl kinase inhibitor, was identified to have robust apoE activity in brain microglia, astrocytes and pericytes. AZ7235 also increased expression of ATP-binding cassette protein A1 (ABCA1), which is involved in the lipidation and secretion of apoE. Moreover, AZ7235 did not exhibit Liver-X-Receptor (LXR) activity and stimulated apoE and ABCA1 expression in the absence of LXR. Target validation studies using *AXL−/−* CCF-STTG1 cells showed that Axl is required to mediate AZ7235 upregulation of apoE and ABCA1. Intriguingly, apoE expression and secretion was significantly attenuated in *AXL*-deficient CCF-STTG1 cells and reconstitution of Axl or kinase-dead Axl significantly restored apoE baseline levels, demonstrating that Axl also plays a role in maintaining apoE homeostasis in astrocytes independent of its kinase activity. Lastly, these effects may require human apoE regulatory sequences, as AZ7235 exhibited little stimulatory activity toward apoE and ABCA1 in primary murine glia derived from neonatal human *APOE3* targeted-replacement mice. Collectively, we identified a small molecule that exhibits robust apoE and ABCA1 activity independent of the LXR pathway in human cells and elucidated a novel relationship between Axl and apoE homeostasis in human astrocytes.

## Background

Alzheimer’s Disease (AD) is the most common form of dementia. Apolipoprotein E (ApoE), which is abundantly secreted from astrocytes, microglia, and pericytes, is the major lipid carrier in the brain [1–3] and the major genetic risk factor for late-onset AD, which accounts for more than 99% of AD cases [4–6]. Humans express three apoE isoforms: apoE2 (protective), apoE3 (neutral) and apoE4 (detrimental), which differ at amino acid positions 112 and 158 and exhibit significant differences in lipid binding, apoE receptor binding, tau-mediated neurodegeneration and amyloid β (Aβ) binding, deposition and clearance [7–10]. Of the three human *APOE* alleles, *APOE4* increases AD risk and reduces age of onset. At least one copy of *APOE4* is present in ~ 17% of the population and ~ 60% of AD patients [5, 11, 12].

As apoE has pleiotropic activities, there is considerable debate about whether raising or lowering apoE leve ls might be beneficial for AD. On one hand, reducing the gene dose of murine *Apoe*, human *APOE3* and human *APOE4* in apoE-targeted replacement mice decreases amyloid burden and tau-mediated neurodegeneration in several AD mouse models [13–16]. Further, intraperitoneal administration of an anti-apoE antibody into AD mice improves cognitive function and reduces brain Aβ load [17], and decreasing *APOE* expression by antisense oligonucleotides significantly alleviates Aβ pathology in amyloid mice homozygous for the *APOE3* or *APOE4* allele [18]. Conversely, in both AD patients and AD animal models, apoE4 protein levels are lower in the central nervous system (CNS) compared to the other apoE isoforms [19–23], and decreased cerebrospinal fluid (CSF) apoE levels are associated with reduced CSF Aβ42 and worse clinical outcome, whereas increased CSF apoE has been suggested to be a protective response to injury in AD [24, 25]. As apoE also plays important roles in lipid transport, neuroinflammation, synaptic plasticity and blood brain barrier (BBB) integrity [26–28], an overall reduction of apoE levels throughout adulthood is not without risk.

A more nuanced approach for apoE-directed therapies for AD may be to modulate apoE functions, which are coupled to apoE’s lipidation status. In the CNS, apoE is directly lipidated by the ATP-binding cassette transporter A1 (ABCA1) protein to form lipoprotein particles that resemble circulating high-density lipoprotein (HDL) in size and density. Both apoE and ABCA1 are transcriptionally regulated by the Liver X Receptor (LXR) and Retinoid X Receptor (RXR) nuclear receptors [29]. Previous studies have used genetic (ABCA1 overexpression) and pharmacologic (LXR or RXR agonists) approaches to show that increased lipidation of apoE lowers amyloid deposition and improves cognitive function in various AD mouse models, whereas decreased apoE lipidation in the absence of ABCA1 exacerbates amyloid pathology [30–33]. Furthermore, targeting unlipidated aggregated apoE using selective antibodies significantly reduced amyloid accumulation in AD mice [34].

To better understand the mechanisms of apoE regulation, as well as the potential therapeutic utility of promoting apoE lipidation, we performed a focused phenotypic screen to identify small molecules that increase apoE secretion from human CCF-STTG1 astrocytoma cells. The screening cascade was carefully designed for facile target identification of novel targets beyond direct LXR agonists. From a library of 14,000 highly annotated small molecules, we identified AZ7235, a previously annotated Axl kinase inhibitor [35], that has robust apoE and ABCA1 activity across several human CNS cell types, independent of LXR activity. Intriguingly, reducing Axl expression significantly attenuated baseline apoE expression in CCF-STTG1 cells, and reconstitution of either wild-type or kinase-dead Axl rescued baseline apoE expression. These effects may depend on human *APOE* regulatory sequences, as AZ7235 had minimal apoE activity and no ABCA1 activity in primary murine glia derived from neonatal human *APOE3* targeted-replacement mice. These novel observations demonstrate that Axl plays an important role in apoE homeostasis in multiple human CNS cell types that are relevant to AD.

## Methods

### Cell models and reagents

Human astrocytoma cell line CCF-STTG1 (cat# 90021502) acquired from European Collection of Authenticated Cell Cultures (ECACC) were expanded at AstraZeneca and used as the parental line to generate an *AXL−/−* line by CRISPR-Cas9 gene editing and polyclonal *AXL*-reconstituted CCF-STTG1 lines (described below). Human microglia clone 3 (HMC3, cat# CRL-3304) was acquired from American Type Culture Collection (ATCC). Immortalized LXR double knockout (LXRα−/LXRβ-) and LXR expressing (LXRα+/LXRβ-) mouse embryonic fibroblasts (MEFs) were kindly provided by Dr. Peter Tontonoz (California, USA) [36]. Primary human astrocytes (cat# 1800) and brain vascular pericytes (cat# 1200) were acquired from ScienCell. Human *APOE3* targeted-replacement mice [37] were obtained from the Cure Alzheimer Fund and primary murine mixed glia were cultured from postnatal day 0–2 pups as described [38]. The LXR agonist T0901317 was acquired from Sigma-Aldrich (cat# 575310). AZ7235 was synthesized as previously described [35]. Commercial Axl inhibitor R428 (cat# 21523) and UNC2025 (cat# 16613) were purchased from Cayman Chemical. Axl inhibitor S49076 was purchased from Selleckchem (cat# S8404). Stocks of all compounds were prepared in dimethyl sulfoxide (DMSO).

### Focused phenotypic screen for apoE secretion

CCF-STTG1 cells were propagated at scale in 10 chamber Cell STACKS (Corning Life Sciences) to generate an assay ready cryobank of 1.6 × 10^9^ cells. Cells were detached using Accutase (Invitrogen/ThermoFisher Scientific cat# 00–4555-56), neutralized with standard media, centrifuged and resuspended in 90% FBS /10% DMSO at a density of 2 × 10^7^ cells/ml and aliquoted for cryopreservation using a controlled rate freezer (Kryo Planer). For the focused screening of the phenotypic compound library, each compound was tested in singlicate at 1 and 10 μM. For validation of primary actives, compounds were tested in a 7-point serial dilution in the 40 nM - 30 μM range in singlicate on two separate occasions.

Cryopreserved CCF-STTG1 cells were carefully thawed in 37 °C water bath, diluted in culture media, and dispensed at a density of 9000 cells/well into 384-well microtitre plates (BD BioCoat Poly D-Lysine, cat#354663) using a Multidrop™ Combi (Thermo Scientific) and standard cassette. Plates were incubated 1 h at room temperature before transferring to standard culture conditions for 18–20 h. Assay-ready compound plates were prepared by dispensing 100 nL compound in DMSO to 384-well polypropylene plates (Greiner, cat# 781220) using an acoustic dispenser (Labcyte, Echo 550 Liquid handler). All plates contained 16 wells each with 100 nL of DMSO (neutral control) or 1 mM of the LXR agonist T0901317 (stimulatory control). The compound plates were further diluted by adding 50 μL of pre-warmed assay medium using a Multidrop™ Combi (Thermo Scientific) and standard cassette. To start the assay, 25 μL of medium was removed from the cell-plate using a Vertical Pipetting Station (Agilent) equipped with a 384-head and replaced by 25 μL from the compound pre-dilution plate. This gave a final assay concentration of stimulatory control T0901317 of 1 μM and a total DMSO concentration of 0.1% in all wells. Cell-plates were incubated at 37 °C, 5% CO_2_ for 65 h, and then 25 μL of the cell-supernatant was removed from the stimulated cell-plate to a new 384 PPV plate (Greiner 781,220) and stored at − 20 °C until analysis by ELISA.

Secreted apoE levels in the focused screen were quantified in a 384-well immunoassay plate (Nunc Maxisorb, cat# 460518) that was prepared by coating with 25 μL/well of a primary anti-human apoE monoclonal antibody, mAb E276 (MabTech, cat# 3712–3-250), diluted to 1.66 μg/mL in PBS. The plates were sealed and placed at 4 °C overnight, followed by washing with 2 × 50 μL wash buffer (PBS with 0.05% Tween-20), and blocking with wash buffer supplemented with 0.1% BSA for 1 h at RT. The blocking solution was removed and the plates were washed with 2 × 50 μL wash buffer before adding 5 μL of the supernatants from stimulated cells together with 20 μL PBST and incubating for 1 h at RT. The plates were then washed with 2 × 50 μL wash buffer and incubated with 20 μL of a biotinylated anti-human apoE monoclonal antibody E887 (MabTech, cat# 3712–6-250), diluted to 0.5 μg/mL in blocking buffer for 1 h. The plates were subsequently washed by 2 × 50 μL wash buffer and incubated with 20 μL streptavidin-HRP (MabTech, cat# 3310–9) diluted 1:1000 in wash buffer supplemented with 0.1% BSA for 1 h. The plates were then washed with 4 × 50 uL wash buffer and 40 μL QuantaBlu Substrate (Thermo, cat# 15162) was added. Fluorescence was measured on a BMG Pherastar plate reader after 1 h. When required, a standard curve of recombinant ApoE3 (MabTech, cat# 3712-1H-6) was included in the ELISA in the range of 31–1000 ng/ml in order to calculate the amount apoE present in cell supernatants. The same apoE ELISA was used in a 96-well plate format for subsequent follow up experiments.

U-2 OS Gal4 chimeric cell-based reporter assays were performed to exclude hit compounds as a direct LXR agonists as previously described [39]. Briefly, the LXR agonist T0901317, used as positive control, and test compounds were added to U-2 OS cells transfected with vectors containing a ligand-binding domain of the LXRα or LXRβ receptor fused to the Gal4 DNA binding domain. Luciferase reporter gene activities were measured after 40 h of treatment.

### Generation of *AXL−/−* CCF-STTG1 cells

Cells were transfected by Neon electroporation (two pulses at 1200 V, 20 ms; Life Technologies), with two custom vectors harboring a CMV-dCas9-ELD-gRNA1 and CMV-dCas9-KKR-gRNA2 of *AXL* containing a guide sequence to *AXL* at exon 1: gRNA1 5′- TCCCTGGGTTGCCCACGAA − 3′ and gRNA2: 5′- GGGACTCACGGGCACCCTT − 3′. Following transfection, single GFP-positive cells were isolated by FACS after 48 h. Clones were screened for indels by PCR amplification of genomic DNA using the following primers: 5′- AAGGACAGGGTGGAACTGAGGGC − 3′ and 5′- TTCCATCACATGCTCAAAGCCGCA -3′. PCR products were Sanger sequenced. Positive *AXL* KO clones was also confirmed by next generation sequencing.

### Knockdown of *AXL* in CCF-STTG1 cells

Two MISSION® siRNAs targeting human *AXL* mRNA NM_001699.5 (Sigma-Aldrich, cat# SIHK0149: 5′-GGUCAUCUUACCUUUCAUGA-3′; SIHK0150: 5′-CUCAGAUGCUAGUGAAGUU-3′) and siRNA Universal Negative Control #1 (Sigma-Aldrich, cat# SIC001) were transfected into CCF-STTG1 cells using Lipofectamine 2000 (ThermoFisher) according to the manufacturer’s instructions. Knockdown efficiency of Axl protein was confirmed by immunoblotting after 72 h of transfection.

### Reconstitution of WT and K567R Axl in *AXL−/−* CCF-STTG1 cells

Axl expression plasmids were generated by BlueSky BioServices (Worcester, MA, USA) who used a human codon-optimized, gene synthesis approach to generate the 2685-bp full-length wild-type human *AXL* cDNA (transcript variant 1, NM_021913) or kinase dead *AXL* (K567R) [40] which was subsequently cloned into the pcDNA3.1 expression vector by GenScript. *AXL−/−* CCF-STTG1 cells were stably transfected with empty expression vector or the above *AXL* constructs using Lipofectamine 3000 (ThermoFisher) according to the manufacturer’s protocol. Four days after transfection, cells were selected with 300 μg/mL G418 (Sigma-Aldrich), and selection was continued for at least 3 weeks before pooling reconstituted stably transfected cells into polyclonal pools.

### Cell culture and treatment

All cells were cultured under standard culture conditions at 37 °C with 95% humidity and 5% v/v CO_2_. CCF-STTG1 cells were cultured in a mixed media consisting of three parts of High-Glucose Dulbecco’s modified Eagle’s medium (DMEM) with L-glutamine (Sigma-Aldrich, cat# D6429) and one part of Ham’s F12 (Sigma-Aldrich, cat# N6658), supplemented with 10% fetal bovine serum (FBS, Gibco) and 1% penicillin/streptomycin (P/S, Gibco). *AXL−/−* and AXL-reconstituted CCF-STTG1 cells were cultured in Roswell Park Memorial Institute medium (RPMI, Gibco) 1640-GlutaMAX™ supplemented with 10% FBS and 1% P/S. Primary murine mixed glia and mouse embryonic fibroblasts were cultured in DMEM (Gibco) supplemented with 10% FBS, 2 mM L-glutamine and 1% P/S. Primary human astrocytes and pericytes were cultured in their respective growth media (containing 2% FBS) provided by ScienCell. HMC3 cells were grown in Eagle’s Minimum Essential Medium (EMEM, ATCC) supplemented with 10% FBS.

For immunoblotting, mRNA, and apoE ELISA assays for follow up experiments, cells were seeded in either 12-well (CCF-STTG1: 200,000 cells/well; MEF: 100,000 cells/well; primary human astrocyte: 300,000 cells/well; primary human pericytes: 100,000 cells/well; HMC3: 100,000 cells/well; primary murine mixed glia: 400,000 cells/well) or 24-well (CCF-STTG1: 100,000 cells/well) plates in their respective standard growth media. After 24 h, cells were treated with fresh growth media containing test compounds at indicated concentration for indicated time intervals.

### Cell viability assay

CCF-STTG1 cell viability was evaluated using CellTiter Blue (resazurin assay; Promega). Cells were incubated with CellTitre Blue as per the manufacturer’s protocol for 1 h and fluorescence was recorded (560_Ex_/590_Em_) using an Infinite 200PRO microplate reader (TECAN). Percent viability is expressed as test compound relative to DMSO control.

### Quantitative RT-PCR

RNA was extracted using the PureLink™ RNA mini-kit (ThermoFisher) according to the manufacturer’s protocol. Real-time quantitative PCR (qRT-PCR) was performed with DNA Green reagents (Roche) on a LightCycler® 96 system (Roche). The qRT-PCR primer sequences used in this study were previously described [39] with additional primers probing mouse *Apoe* (Forward: 5′- AACCGCTTCTGGGATTACCT-3′; Reverse: 5′-TGTGTGACTTGGGAGCTCTG-3′). Each sample was assayed at least in duplicate and normalized to glyceraldehyde 3-phosphate dehydrogenase (GAPDH) (human) or β-actin (mouse).

### Electrophoresis and Immunoblotting

For denaturing polyacrylamide gel electrophoresis (PAGE), cells were washed with 1x phosphate buffered saline (PBS) and lysed in radioimmunoprecipitation assay (RIPA) lysis buffer (20 mM Tris, 1% NP40 Alternative, 5 mM ethylenediaminetetraacetic acid (EDTA), 50 mM NaCl, 10 mM Na pyrophosphate, 50 mM NaF, and complete protease inhibitor (Roche), pH 7.4). Protein concentration was determined by BCA Protein Assay Kit (Pierce). Cellular proteins (20–40 μg/well) were mixed with loading dye with a final concentration of 2% sodium dodecyl sulfate (SDS) and 1% β-mercaptoethanol, incubated for 5 min at 95 °C and resolved on house-made 10% Tris-HCl polyacrylamide gels or pre-casted NuPAGE™ 4–12% Bis-Tris Protein Gels (Thermo Fisher). Samples derived from the same experiment were always run on the same gel. Proteins were transferred onto polyvinylidene difluoride (PVDF, Millipore) membranes at 24 V overnight at 4 °C. To minimize gel-to-gel variation, blots were typically cut into strips to probe for multiple proteins of distinct molecular weights that were separated on the same gel and preserve a common loading control for each protein of interest. After blocking with 5% non-fat milk in PBS for 1 h, membranes were probed for 1 h at 4 °C with 1:1000 rabbit-anti-apoE (Cell Signaling Technology, cat# 13366), 1:1000 monoclonal rabbit-anti-ABCA1 [Clone 1276B] (Novus, cat# NBP2–54792), 1:1000 rabbit-anti-Axl (Cell Signaling Technology, cat# 8661). Blots of the same experiment were stripped with 1x ReBlot Plus Mild Antibody Stripping Solution (Millipore, cat# 2502), re-blocked and re-probed for 30 min with 1:5000 anti-glyceraldehyde 3-phosphate dehydrogenase (GAPDH) or anti-β-actin (Millipore) as loading controls. Membranes were washed with PBST (1x PBS with 0.05% Tween-20) and then incubated for 1 h with horseradish peroxidase (HRP)-labeled anti-mouse or anti-rabbit secondary antibodies (Jackson Immuno-Research). For native PAGE, media samples were mixed with non-denaturing loading dye to a final concentration of 0.04% bromophenol blue, 4.0% glycerol, and 100 mM Tris (pH 6.8) and resolved on 6% non-denaturing Tris-HCl polyacrylamide gels. Native gels were then transferred as described above and probed with 1:1000 rabbit-anti-apoE antibody (Cell Signaling Technology, cat# 13366) overnight. Results were visualized using chemiluminescence (ECL, Amersham) and blot images were captured on xfilm or with a Bio-Rad ChemiDoc MP Imaging System (Bio-Rad). Band density was quantified using ImageJ software (version 1.47q, National Institutes of Health).

### Cholesterol efflux assay

CCF-STTG1 cells were seeded at 100,000 cells/well in 24-well plates and cultured for 24 h before labeling for 24 h with 1 μCi/ml of ^3^H-Cholesterol (PerkinElmer Life Sciences) in growth media supplemented with DMSO, 1 μM T0901317, or 1 μM AZ7235. Labeled cells were then washed and equilibrated in serum-free media for 60 min. Serum-free media containing the same compound treatments were then added to the cells in the absence (NA, no acceptor) or presence of 10 μg/ml of exogenous lipid-free apoA-I (a kind gift from CSL Behring, Switzerland). After 24 h at 37 °C, culture media was collected and cells were lysed by addition of 0.2 M NaOH and 0.2% Triton-X, followed by incubation at room temperature for a minimum of 1 h. Radioactivity in media and cell lysate samples was quantified by scintillation counting (PerkinElmer). The percent cholesterol efflux was calculated as the total counts per minute (CPM) in the media divided by the sum of the CPM in the media plus in the cell lysate.

### LXR reporter luciferase assay

To interrogate LXR activation in CCF-STTG1 cells, the LXRα Cignal Reporter Assay kit (Qiagen) was used according to the manufacturer’s instructions. The LXRα reporter system is a mixture of a LXRα-responsive Firefly luciferase construct and a constitutively expressing Renilla luciferase construct (40:1) under the transcriptional control of a minimal CMV promoter and tandem repeats of LXR-response elements (LXRE). CCF-STTG1 cells were transfected with expression vectors containing WT or mutant LXRα without LXRE, followed by treatment with either 1 μM T0901317 or 3 μM AZ7235 24 h post transfection. LXRα activity was monitored by a dual luciferase assay (Promega) 24 h post treatment and luciferase activities were read on Tecan Spark Multimode Microplate Reader.

### Statistical analysis

Statistical analyses were performed using randomized block ANOVA with experimental runs as blocks to minimize inter-experimental variation [41]. For immunoblot analysis, raw densitometry data (target protein value over loading control protein value) were first log transformed and then analyzed by a two-way (“Experiment” and “Drug Treatment” as the two factors) ANOVA model with “Experiment” being the blocking factor and with a Dunnett’s multiple comparison post-test (i.e. each drug treatment condition compared to vehicle control). For qRT-PCR analysis, ΔC_T_ values (target gene C_T_ minus reference gene C_T_) were used in the same two-way ANOVA model with “Experiment” being the blocking factor. For cholesterol efflux and MEF LXR-dependency experiments, a three-way (“Drug”, “Experiment” and “ApoA-I treatment/genotype” as the three factors) ANOVA was used, where “Experiment” was used as the blocking factor and Sidak’s multiple-comparison tests were used to compare either the test compounds’ effect over vehicle control within each genotype/treatment condition, or the effect of genotype/treatment themselves under each test compound condition.

For assays using arbitrary units, such as immunoblot and qRT-PCR, results are plotted as fold-change over vehicle control (dotted lines) ± 95% confidence interval (i.e. the mean difference of treatment group versus control group calculated from the aforementioned ANOVA analysis) of the indicated number of independent experiments. For assays using standard units, such as ELISA, cholesterol efflux and LXR luciferase assay, data are presented as mean measurement ± standard deviation from the indicated number of experiments. All statistical analyses were performed using Statistical Package for the Social Sciences (SPSS) (version 23) and *P*-values < 0.05 were considered significant. Prism 6 (GraphPad Software) was used to graph all data.

## Results

### Phenotypic screening of apoE upregulation in CCF-STTG1 cells identifies AZ7235

We tested ~ 14,000 structurally diverse compounds for their ability to increase apoE levels in the media overlaying CCF-STTG1 cells. As previously described [42], the compound collection included marketed drugs, internal AstraZeneca compounds, and literature compounds with a high degree of compound annotation and activity values of less than 100 nM against more than 1500 biological targets including GPCRs, nuclear hormone receptors, ion channels, and enzymes. A focused screen at 1 and 10 μM identified 419 compounds that were active at both concentrations (2.8% hit-rate) whereas 225 were active only at 1 μM and 561 only at 10 μM. A total of 1000 compounds were selected for confirmation in concentration response screening and 717 compounds were indeed confirmed as active. For the 585 of these compounds with no prior data on activity versus LXR, counter screening was performed in an LXR reporter gene assay. After filtering out all compounds that were known to be, or identified as, active on unwanted or intractable targets such as the LXR receptors, the remaining 235 compounds were further analyzed based on their annotated molecular targets. We selected AZ7235, a previously described Axl receptor tyrosine kinase (RTK) inhibitor [35], for follow up studies based on the potency of the initial hit, and the potential biological interest of the annotated target.

### AZ7235 upregulates apoE and ABCA1 expression in multiple apoE-secreting human CNS cell types

**Fig. 1:**
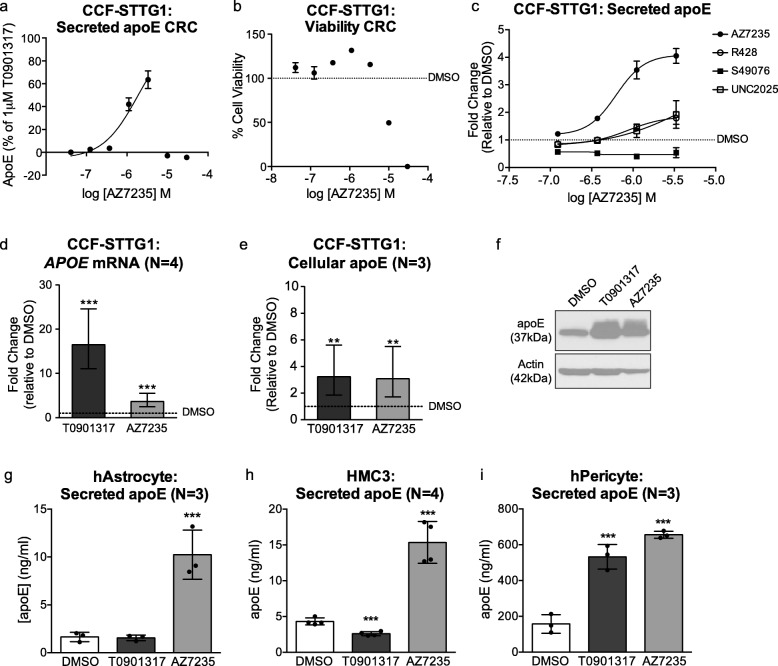
AZ7235 increases apoE expression in various CNS cell types. (a-b) CCF-STTG1 cells were treated with AZ7235 using a 7-point concentration response curve at the indicated concentration (0.041–30 μM) for 72 h. (**a**) ApoE secretion was measured by ELISA and data are expressed as % apoE secretion relative to DMSO (0%) and 1 μM of the positive control LXR agonist T0901317 (100%). (**b**) Cell viability was measured by CellTiter-Blue assay and data are expressed as percentage change relative to DMSO treatment (100%, dashed line). (**c**) Log dose response curves for other Axl inhibitors R428, S49076 and UNC2025 (0.1 μM – 3 μM) on apoE secretion in CCF-STTG1 after 72 h treatment. Data are expressed as fold-change relative to DMSO treatment (dashed line). Error bars represent range of duplicate wells in one representative assay. (**d**) *APOE* mRNA levels were measured by qRT-PCR and (**e**) cellular apoE protein levels were measured by immunoblot in CCF-STTG1 cells after 72 h treatment with vehicle control DMSO, 1 μM T0901317, 3 μM AZ7235. (f) Representative immunoblot of cellular apoE. Images were cropped to show relevant lanes. Graphs of (**d**) and (**e**) represent fold-change over DMSO control (dashed line) and +/− 95% confidence intervals from N independent experiments indicated in brackets. (**g**-**i**) Secreted apoE levels were measured in primary human astrocytes (**g**), HMC3 (**h**) and primary human brain vascular pericytes (**i**) after 72 h drug treatment. Graph represents mean concentration and standard deviation from N independent experiments indicated in brackets. ** P < 0.01, *** P < 0.001 compared to vehicle control using blocked two-way analysis of variance (ANOVA) post-hoc tests

We first confirmed that AZ7235 dose-dependently increased ApoE secretion up to concentrations of 10 uM (Fig. 1a), above which the compound was cytotoxic resulting in a non-specific decline in ApoE secretion (Fig. 1b). To determine whether other Axl kinase inhibitors also regulate apoE secretion from CCF-STTG1 cells, we evaluated R428 [43], S49076 [44], and UNC2025 [45] in a concentration response assay. Compared to AZ7235, R428 and S49076 exhibited modest apoE activity whereas UNC2025 had minimal effect on the apoE response. (Fig. 1c). We also tested whether CCF-STTG1 cells respond to up to 1000 ng/ml of Gas-6, a well-established Axl ligand [40] and found no response (data not shown), suggesting that AZ7235 may use a non-canonical mechanism to regulate apoE in human CCF-STTG1 cells, potentially explaining the differential activities of these Axl inhibitors on apoE secretion.

In CCF-STTG1 cells, AZ7235 significantly induced apoE expression at both mRNA (Fig. 1d) and cellular protein levels (Fig. 1e-f) after 72 h at 3 μM, the maximum effective concentration. AZ7235 also exhibited robust apoE activity in other apoE-secreting CNS cell types including primary human astrocytes, HMC3 microglia cells, and primary human brain vascular pericytes **(**Fig. 1g-i**).** Intriguingly, primary human astrocytes and HMC3 cells were unresponsive to T0901317, demonstrating cell type differences in the regulation of apoE expression.

**Fig. 2:**
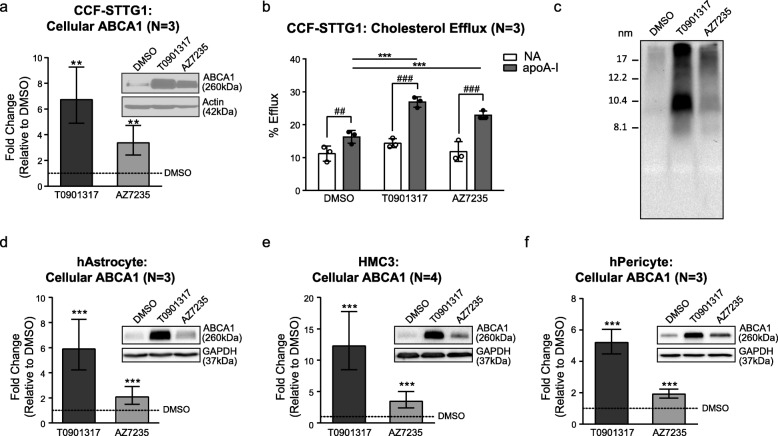
AZ7235 increases ABCA1 expression. (**a**) Cellular ABCA1 protein levels were measured by immunoblot in CCF-STTG1 cells after 72 h treatment with DMSO, 1 μM T0901317, or 3 μM AZ7235. Representative blot was generated from same gel shown in Fig. 1f. (**b**) CCF-STTG1 cells were labeled with ^3^H-cholesterol with co-treatment of DMSO alone, 1 μM T0901317, or 3 μM AZ7235 for 24 h. Cholesterol efflux over 24 h in the absence (NA) or presence of 10 μg/ml of lipid-free apoA-I along with the above drug treatment was evaluated. Graphs represent mean % efflux and standard deviation of three independent experiments. * P < 0.05, ** P < 0.01, *** P < 0.001 comparing drug effect over respective DMSO control; ### P < 0.001 comparing between NA vs. apoA-I within each drug condition by blocked three-way ANOVA post-hoc tests. (**c**) Particle size distribution of apoE-containing lipoproteins in the unconcentrated 72-h-conditioned media from drug-treated CCF-STTG1 were assessed by 6% native PAGE followed by immunoblotting for apoE. The ladder on the left represents Stokes diameter. (**d**-**f**) Cellular ABCA1 protein levels were measured by immunoblot in primary human astrocytes, HMC3 and primary human brain vascular pericytes after 72 h treatment with vehicle control DMSO, 1 μM T0901317, 3 μM AZ7235. Graphs represent fold-change over DMSO control (dashed line) and +/− 95% confidence intervals from N independent experiments indicated in brackets. *** P < 0.001 compared to vehicle control using blocked two-way ANOVA post-hoc tests. Immunoblot images were cropped to show relevant lanes

As both apoE levels and function are related to its lipidation by ABCA1, we examined the effect of AZ7235 on ABCA1 in CCF-STTG1 cells. We found that AZ7235 significantly increased both ABCA1 protein levels (Fig. 2a) and ABCA1 activity as measured by a cholesterol efflux assay to exogenous apoA-I (Fig. 2b). To examine whether the particle size distribution of the apoE-containing lipoproteins secreted by CCF-STTG1 cells was affected by compound treatment, conditioned media was resolved by non-denaturing gel electrophoresis, revealing that, similar to T0901317, AZ7235 increased the amount of HDL-like sized apoE particles ranging from ~ 8 to 17 nm in diameter (Fig. 2c). These results demonstrate that AZ7235 not only upregulates apoE expression but also increases ABCA1 protein levels and activity, resulting in enhanced production of native lipidated apoE particles that are secreted from CCF-STTG1 cells. We also confirmed that AZ7235 increased ABCA1 protein levels in primary human astrocytes, HMC3, and primary brain vascular pericytes (Fig. 2d-f).

Overall, these data demonstrate that AZ7235 robustly stimulates apoE and ABCA1 expression and activity across multiple apoE-secreting CNS cell types from human origins.

### AZ7235 shows no direct or indirect activation of the LXR pathway

**Fig. 3:**
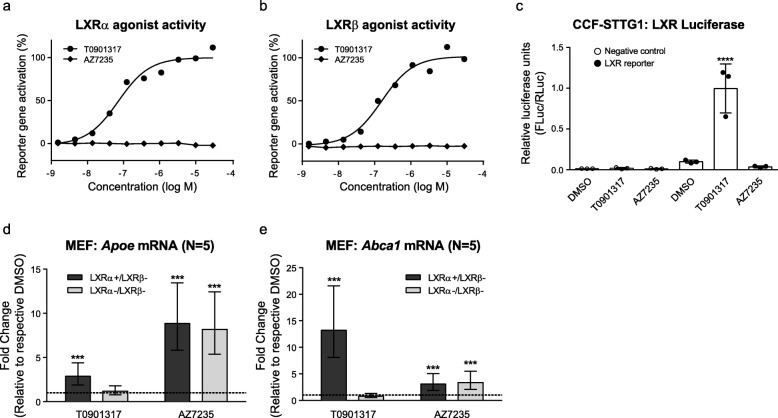
AZ7235-mediated apoE and ABCA1 induction is LXR-independent. U2-OS LXR-Gal4 chimeric Luciferase reporter assays were used to demonstrate that AZ7235 (1.5 nM – 30 μM) have no direct LXRα (**a**) or LXRβ (**b**) agonist activity, unlike the direct LXR agonist T091317, after 40 h of treatment. (**c**) Vehicle control, DMSO, 1 μM T0901317, 3 μM AZ7235 were added to CCF-STTG1 cells transfected with a mixture of LXR-responsive Firefly luciferase construct and constitutively expressing Renilla luciferase construct as an internal control. Luciferase activities were measured after 24 h of treatment. Error bars represent standard deviation from technical triplicates. *** P < 0.0001 by ANOVA post-hoc analysis. (**d**-**e**) LXR-knockout (LXRα−/β-) and LXRα-expressing (LXRα+/β-) MEF cells were treated with DMSO or drugs for 48 h. *Apoe* and *Abca1* mRNA levels were measured by qRT-PCR. Graph represents fold-change over respective DMSO control (dash line) +/− 95% CI from 5 experiments

Several approaches were used to test whether AZ7235 has direct or indirect LXR agonist activity. First, a GAL4 chimeric reporter-based assay in U-2 OS cells showed that while T0901317 exhibited strong agonist activity for both the LXRα and LXRβ receptors in a concentration-dependent manner as expected, AZ7235 showed no activity at either LXR receptor (Fig. 3a-b), indicating that AZ7235 does not directly bind to LXR receptors. Using the same chimeric reporter constructs, lack of direct nuclear receptor activity was confirmed using a receptor binding assay at 0.3 and 3 μM AZ7235 for several additional orphan nuclear receptors including RXRα, RXRβ, RXRγ, peroxisome proliferator-activated receptor γ (PPARγ), and farnesoid-X-receptor (FXR), all of which were below the threshold of ≥2-fold activation (data not shown). LXR reporter luciferase assays were then used to test whether AZ7235 indirectly activates the LXR pathway. While T0901317 exhibited strong LXR activity at 1 μM, AZ7235 showed no activity at 3 μM, demonstrating that this compound also does not indirectly stimulate the LXR pathway (Fig. 3c). Finally, immortalized mouse embryonic fibroblasts (MEFs) deficient for both LXRα and LXRβ and isogenic MEFs reconstituted with LXRα were used to evaluate AZ7235 activity on *Apoe* and *Abca1* expression. As expected, *Apoe* and *Abca1* upregulation induced by T0901317 was completely abolished in LXRα and LXRβ double knockout MEFs. However, the activity of AZ7235 on *Apoe* and *Abca1* induction was unchanged in the absence of LXR (Fig. 3d-e). Taken together, these studies demonstrate that the mechanism of action by which AZ7235 increases apoE and ABCA1 expression is independent of LXR signaling.

### Axl is required for AZ7235 activity

**Fig. 4:**
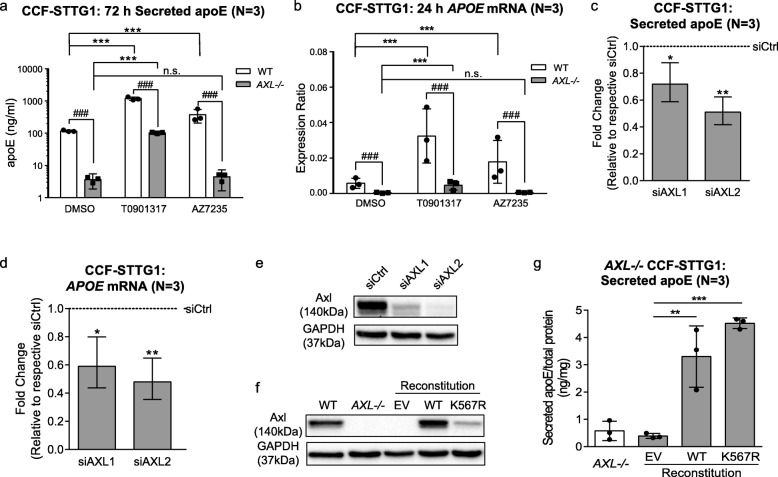
Axl is the target of AZ7235 and plays a role in regulating apoE homeostasis. (**a**) Secreted apoE and (**b**) *APOE* mRNA levels were measured in WT and *AXL−/−* CCF-STTG1 cells after 72 h treatment with vehicle control DMSO, 1 μM T0901317 or 3 μM AZ7235. (**c**) Secreted apoE and (**d**) *APOE* mRNA were measured in CCF-STTG1 cells after transfection with siRNAs targeting *AXL* for 72 h. (**e**) Representative immunoblot of Axl after siRNA transfection. Graphs represent fold-change over scrambled siRNA control (dashed line) and +/− 95% confidence intervals from 3 independent experiments. (**f**) Representative immunoblot of Axl in WT and K567R Axl stable-expressing *AXL−/−* CCF-STTG1 cells. Immunoblot images were cropped to show relevant lanes. (**g**) Secreted apoE levels were measured in *AXL−/−* CCF-STTG1 stably transfected with empty vector (EV), WT or K567R Axl. Error bars represent standard deviation. * P < 0.05, ** P < 0.01, *** P < 0.001 compared to vehicle control using blocked ANOVA post-hoc tests. ### P < 0.001 comparing between WT vs. *AXL*-/- within each drug condition

AZ7235 is a type-I inhibitor of the Axl RTK with a previously reported IC50 of 5 nM [35]. As type I kinase inhibitors often have multiple targets, we determined whether the apoE activity of AZ7235 requires Axl. We used CRISPR (clustered regularly interspaced short palindromic repeats) to knock out *AXL* from CCF-STTG1 cells. We found that without *AXL*, AZ7235 had no effect on apoE secreted protein or mRNA expression, whereas T0901317 retained its ability to upregulate apoE (Fig. 4a-b).

### Axl regulates apoE homeostasis in CCF-STTG1 cells

Intriguingly, we observed that baseline levels of apoE mRNA and secreted protein were significantly reduced in *AXL−/−* CCF-STTG1 cells when comparing DMSO treated wild-type and *AXL* knock-out CCF-STTG1 cells (Fig. 4a-b), raising the hypothesis that Axl is associated with regulation of apoE homeostasis. To determine whether this effect was specific to *AXL*, or an off-target result of the CRISPR editing, we used siRNA to knockdown *AXL* in CCF-STTG1 cells. Experiments using two distinct siRNAs showed that reduced *AXL* expression also significantly lowered baseline apoE levels (Fig. 4c-e), mimicking the phenotype of *AXL−/−* CCF-STTG1 cells. We then reconstituted *AXL−/−* CCF-STTG1 cells with plasmids expressing either wild-type (WT) Axl or a kinase-dead version of Axl carrying the K567R mutation that abrogates tyrosine kinase activity and generated stable polyclonal cell lines for each construct (Fig. 4f). Compared to the negative control transfected with empty vector (EV), baseline apoE levels were elevated in both reconstituted *AXL−/−* CCF-STTG1 lines (Fig. 4g), demonstrating that Axl affects astrocyte apoE homeostasis independent of its kinase activity.

### AZ7235 has little activity on apoE and ABCA1 in primary murine glia from targeted-replacement *APOE3* mice

**Fig. 5:**
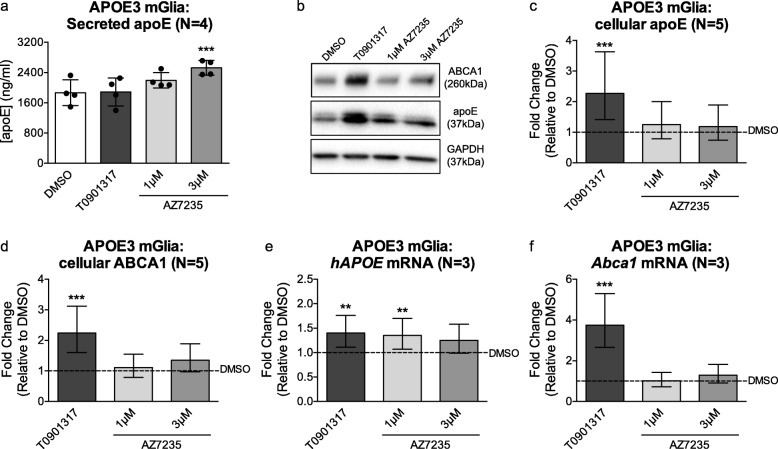
AZ7235 has nominal effects on secretion and mRNA levels of apoE and no effect on ABCA1 expression in primary murine mixed glia expressing human apoE3. Primary mixed glial cells (~ 90% astrocytes, 10% microglia) were cultivated from neonatal human *APOE3* targeted-replacement mice. Cells were re-plated at 18–20 days in vitro followed by treatment with vehicle (DMSO), 1 μM T0901317, 1 μM and 3 μM AZ7235 for 72 h. (**a**) Secreted apoE levels in conditioned media were measured by apoE ELISA. The graph represents mean concentration and standard deviation from 4 independent experiments. (**b**) Representative immunoblot of cellular ABCA1 and apoE with GAPDH as the loading control. Immunoblot images were cropped to show relevant lanes. (**c**) Quantification of cellular apoE and (**d**) ABCA1 protein levels in murine glia lysates by immunoblotting. (**e**-**f**) Human *APOE* (**e**) and mouse *Abca1* (**f**) mRNA levels were measured by qRT-PCR. Graphs represent fold-change over DMSO control (dashed line) and +/− 95% confidence intervals from N independent experiments indicated in brackets. ** P < 0.01, *** P < 0.001 compared to vehicle control by blocked ANOVA post-hoc tests

To investigate the activity of AZ7235 in mouse cells, we cultivated primary murine glia derived from neonatal human *APOE3* targeted-replacement mice as previously described [38]. Cells were re-plated at 18–20 days in vitro followed by treatment with DMSO, 1 μM T0901317, 1 μM and 3 μM AZ7235 for 72 h. Unlike in human cells, AZ7235 had greatly reduced activity on apoE and ABCA1 modulation in murine glia that express the human apoE3 protein. Specifically, 3 μM of AZ7235 led to a 1.5-fold, statistically significant increase in secreted apoE from murine mixed glia (Fig. 5a), which is far less than the 4–6 fold increase in secreted apoE in CCF-STTG1, human primary astrocytes and primary human pericytes at the same concentration of AZ7235. Again contrasting with human cells, neither the cellular protein levels of apoE nor ABCA1 were increased by AZ7235 in murine glia (Fig. 5b-d). Although the LXR agonist T0901317 significantly increased cellular apoE and ABCA1 protein levels by 72 h in primary mixed glia, T0901317 treatment did not increase secreted apoE levels in these cells. Further, although AZ7235 robustly upregulates both apoE and ABCA1 mRNA in multiple human cell types (Fig. 1), AZ7235 led to modest, less than 1.5-fold, increase in *APOE* mRNA (Fig. 5e) and no effect on *Abca1* mRNA levels in primary murine glia (Fig. 5f). These data suggest that apoE is differentially regulated in murine compared to human cells, as the targeted replacement mice used in this study have humanized *APOE* introns between exons 2–3 and exons 3–4 but retain the murine *Apoe* 5′ and 3′ untranslated regions.

## Discussion

ApoE plays an undeniably important yet enigmatic role in AD pathogenesis and remains a challenging drug target. The present study was designed to provide new insights into apoE regulation that may lead to novel therapeutic approaches targeting apoE levels and lipidation. We identified compound AZ7235 as a modulator of apoE expression in CCF-STTG1 cells. AZ7235 also stimulated apoE secretion in multiple other CNS cell types including HMC3 microglial cells, primary human astrocytes and brain vascular pericytes. As AZ7235 also induced ABCA1 activity, the resultant apoE particles are lipidated and resemble native apoE particles in size.

Importantly, AZ7235 is neither a direct LXR agonist nor indirectly stimulates LXR activity, distinguishing AZ7235 from other compounds returned from our previous efforts that identified several compound classes that robustly stimulate both apoE and ABCA1 expression in astrocytes via the LXR-mediated pathway [39, 46, 47]. A recent study identified Ondansetron, an FDA-approved 5-HT3 antagonist, as an apoE modulator that also involves the LXR pathway [48]. As LXR activation is undesirable due to hepatotoxic side effects, several studies have aimed to identify LXR-independent pathways that stimulate apoE expression and these have returned inhibitors of 3β-hydroxysterol Δ(24)-reductase, 7-dehydrocholesterol reductase, and pan class I histone deacetylases [49, 50], lending support that apoE production and lipidation can be regulated though LXR-independent pathways in the CNS.

AZ7235 is from a distinct class of apoE modulators that targets Axl, an RTK that belongs to the TAM (TYRO3, Axl, and MERTK) family. Axl is ubiquitously expressed in a wide range of tissues and plays important roles in apoptosis, immune response, cytokine secretion, cell proliferation and survival. It is overexpressed in various cancers including breast, pancreatic and lung and is associated with low survival rates [51, 52]. The role of Axl in the CNS has gained recent attention as Axl levels may be a potential biomarker for brain damage [53–55]. In addition to the identification of AZ7235 as a novel apoE modulator, our study demonstrates that Axl also plays a pivotal role in apoE homeostasis in CCF-STTG1 cells. Specifically, knock-out and knock-down experiments show that reducing Axl levels significantly reduces baseline levels of apoE mRNA and secreted protein, whereas reconstitution of Axl in *AXL*-deficient cells significantly elevates baseline apoE expression. Furthermore, Axl kinase activity is not required to regulate baseline apoE levels, as reconstitution of kinase-dead K567R Axl [40, 56, 57] also significantly elevates baseline apoE levels in Axl-deficient CCF-STTG1 cells. To our knowledge, this is the first study to link Axl to apoE regulation in astrocyte cells.

This new insight may be highly relevant to the role of apoE in inflammation, where several recent studies demonstrate a role for Axl in brain inflammatory cells. For example, microglia isolated from aged mouse brain display significantly elevated Axl expression [58]. Aβ plaque-associated microglia from transgenic AD mice challenged with LPS have a proinflammatory phenotype with significantly increased Axl expression [59]. Importantly, Axl plays a major role in microglial phagocytosis [60] and has been demonstrated to be expressed on plaque-associated microglia, promoting plaque clearance [61]. In addition, Axl and apoE are members of a cluster of genes believed to mediate a phenotypic switch from homeostatic microglia to neurodegenerative, disease-associated microglia [62]. Our present study is the first to describe a novel relationship between Axl and apoE homeostasis in astrocytes. Additionally, we also show that AZ7235 also stimulates apoE expression and secretion in microglia and pericytes. Further studies will be needed to interrogate the mechanisms and functional significance of the Axl-apoE signaling axis in CNS cells types that produce apoE.

Further support for a non-canonical mechanism linking Axl to apoE secretion stems from two observations. First, we found a disconnect between the in vitro inhibition of Axl kinase activity and apoE expression or secretion across the Axl inhibitors we tested. Second, we observed a lack of apoE response after Gas6 stimulation in these cells. Further studies will be needed to elucidate the non-canonical mechanism by which AZ2735 acts to stimulate apoE.

Importantly, we also observed that AZ7235 has little to no effect on apoE and ABCA1 in murine glial cells, even when these cells express human apoE protein. The human APOE targeted-replacement mice we used to cultivate primary glial cells were generated using a knock-in strategy that retains human introns between exons 2–3 and 3–4 but retains the murine 5’UTR and 3’UTR sequences [37]. Murine apoE is known to be much less responsive to known apoE regulators such as GW3965 [32] and Bexarotene [63], thus we expected a reduced signal in primary murine glia. As expected, AZ7235 only mildly upregulates apoE in murine glia at both the transcriptional and secretion levels, whereas the compound has no effect on murine ABCA1 expression. Although these observations raise questions about the suitability of existing mouse models for further research using AZ7235, they also establish a rationale to develop new humanized apoE mouse models that express human apoE isoforms from human regulatory sequences.

## Data Availability

All data generated or analyzed in this study are included in this published article.

## Abbreviations

AD: Alzheimer’s disease
ATCC: American type culture collection
ANOVA: Analysis of variance
apoE: Apolipoprotein E
ABCA1: ATP binding cassette transporter A1
BBB: Blood brain barrier
CSF: Cerebrospinal fluid
CNS: Central nervous system
CPM: Counts per minute
DMSO: Dimethyl sulfoxide
DMEM: Dulbecco’s modified Eagle’s medium
EV: Empty vector
ELISA: Enzyme linked immunosorbent assay
EDTA: Ethylenediaminetetraacetic acid
FXR: Farnesoid-X-receptor
FBS: Fetal bovine serum
GAPDH: Glyceraldehyde 3-phosphate dehydrogenase
HDL: High density lipoprotein
HTS: High throughput screen
HRP: Horseradish peroxidase
HMC3: Human microglia clone 3
LXR: Liver X receptor
LXRE: LXR-response elements
MEF: Mouse embryonic fibroblasts
PPARγ: Peroxisome proliferator-activated receptor γ
P/S: Penicillin/streptomycin
PAGE: Polyacrylamide gel electrophoresis
PVDF: Polyvinylidene difluoride
PBS: Phosphate buffered saline
qRT-PCR: Real-time quantitative PCR
RTK: Receptor tyrosine kinase
RXR: Retinoid X receptor
RIPA: Radioimmunoprecipitation assay
RPMI: Roswell Park Memorial Institute
SDS: Sodium dodecyl sulfate
SPSS: Statistical Package for the Social Sciences
